# Effect of FTO on cardiac hypertrophy through the regulation of *OBSCN* expression

**DOI:** 10.1016/j.gendis.2023.101165

**Published:** 2023-11-11

**Authors:** Lili Chen, Yiheng Zhao, Wenjing Wang, Shuchen Zhang, Xiang Zhou

**Affiliations:** a Central Laboratory, The Second Affiliated Hospital of Soochow University, Suzhou, Jiangsu 215004, China; b Department of Cardiology, The Second Affiliated Hospital of Soochow University, Suzhou, Jiangsu 215004, China; c Intensive Care Unit, The Second Affiliated Hospital of Soochow University, Suzhou, Jiangsu 215004, China

*N*^6^-methyladenosine (m^6^A) RNA methylation exerts significant functions in regulating various cardiovascular diseases. Following the recognition of the first m^6^A demethylase, namely fat and obesity-associated protein (FTO), mounting evidence has shown that polymorphism of the *FTO* gene leads to an increased occurrence of cardiovascular-related risk factors including obesity, diabetes, and inflammation.^1^ As shown previously, FTO is directly involved in the physiological processes of hypertension, ischemic cardiomyopathy, and heart failure, and it could be a pathogenic factor and potential therapeutic target for various cardiovascular diseases.^2^ It is experimentally confirmed that m6A deficiency leads to abnormal cardiac function in mice; m6A-deficient mice are more inclined to develop cardiac dysfunction stimulated by serum-induced myocardial hypertrophy.^3^ Therefore, m^6^A modification is required to maintain cardiac homeostasis. FTO ameliorates ischemia-induced cardiac systolic dysfunction, which is induced by the demethylating effect of FTO, leading to an inhibition of degrading cardiac contractile transcripts and an enhanced expression of transcripts in the ischemic state.^4^ These researches showed that FTO might be pivotal in various cardiovascular diseases, particularly in cardiac hypertrophy, by demethylation. However, the specific mechanism of action remains unelucidated. Here, we investigated whether FTO regulates cardiac hypertrophy and the underlying mechanism of its regulatory effect.

As a common induction method of cardiomyocyte hypertrophy, different concentrations of angiotensin II (Ang II) were used to treat cardiomyocytes. We found that compared with 0 μM Ang II-treated cardiomyocytes, 1 μM Ang II-treated cardiomyocytes had obviously increased mRNA expression of myocardial hypertrophy indices (β-MHC/ANP/Serca-2a) (Fig. S1A). m^6^A quantification experiment (Fig. 1A) and immunofluorescence detection of m^6^A antibodies (Fig. S1B) revealed that Ang II reduced the m^6^A methylation level in cardiomyocytes. To fully explore the underlying role of m^6^A methylation and to further identify dysregulated m^6^A methylases in myocardial hypertrophy, the expression of several m^6^A methylases was detected, including m^6^A reader proteins (YTHDF1, YTHDF2, and YTHDC1), methyltransferases (METTL3 and METTL14), and demethylases (FTO and ALKBH5) in myocardial tissues or cardiomyocytes of mice. Western blotting assays showed that the protein expression of FTO was increased in the myocardial tissue of transverse aortic constriction (TAC)-treated mice and in hypertrophic cardiomyocytes stimulated with Ang II; in contrast, the expression of other methylases was not consistent in mouse myocardial tissues or cardiomyocytes (Fig. S2A). qPCR also showed that treatment with 1 μM Ang II increased the mRNA expression of hypertrophy indices (β-MHC/ANP/Serca-2a) and FTO, but not that of other m^6^A methylases; this finding was consistent with the levels of protein expression (Fig. S2B). Therefore, we chose FTO, a demethylase, as the crucial methylase in myocardial hypertrophy for further research. These results revealed that m^6^A RNA methylation in cardiomyocytes is affected by FTO.

**Figure 1:**
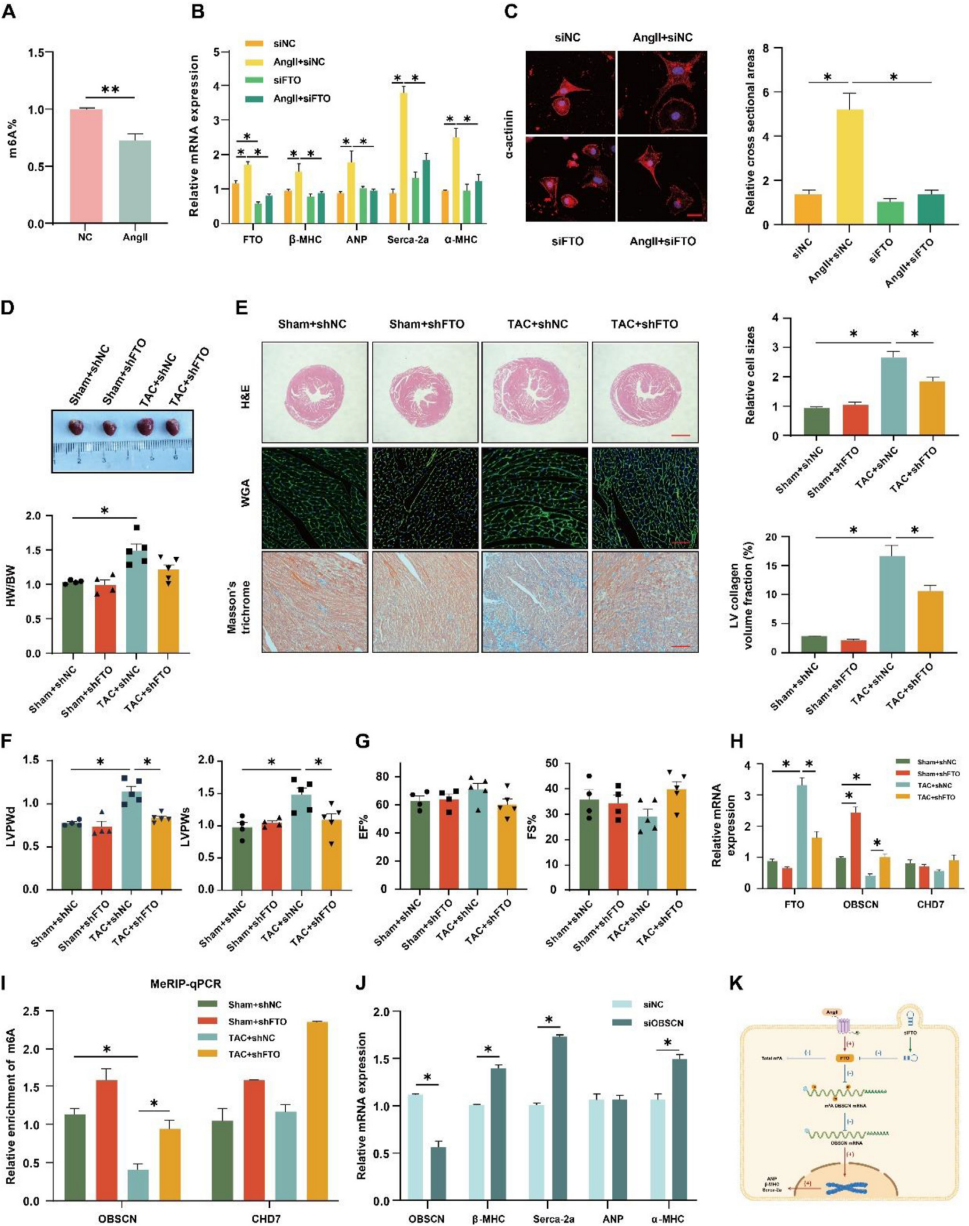
FTO knockdown alleviated cardiac hypertrophy through the regulation of OBSCN expression. **(A)** m^6^A RNA methylation quantification by the colorimetric method. **(B)** Relative mRNA levels of FTO, β-MHC, ANP, Serca-2a, and α-MHC in cardiomyocytes induced by 1 μM Ang II and/or transfected with siFTO. **(C)** Immunofluorescence detection of α-actinin and relative cross-sectional areas in cardiomyocytes induced by 1 μM Ang II and/or transfected with siFTO. Scale bar, 20 μm. **(D)** Mouse heart images and heart-to-body weight ratio (HW/BW) were measured. **(E)** Left: Representative histological images of H&E, WGA, and Masson's trichrome-stained heart sections of mice. Scale bar, 1 mm, 20 μm, and 20 μm, respectively. Right: Relative cell size and LV collagen volume fraction were quantified in immunohistochemical images. **(F, G)** LVPWd, LVPWs, EF%, and FS% of mice were determined by echocardiography. **(H)** Relative mRNA expression of FTO, OBSCN, and CHD7 in myocardial tissues of mice was detected by qPCR. **(I)** Relative m^6^A methylation levels of OBSCN and CHD7 in myocardial tissues of mice were detected by MeRIP-qPCR. **(J)** Relative mRNA levels of OBSCN, β-MHC, ANP, Serca-2a, and α-MHC in cardiomyocytes transfected with siOBSCN. **(K)** FTO down-regulates the mRNA expression of the OBSCN gene by demethylation, leading to cardiac hypertrophy. ^∗^*P* < 0.05; ^∗∗^*P* < 0.01. LV, left ventricular; LVPWd, LV posterior wall thickness at end-diastole; LVPWs, LV posterior wall thickness at end-systole; EF, ejection fraction; FS, fractional shortening.

To explore the potential effect of FTO on hypertrophy development, we determined the effect of FTO on cardiomyocyte hypertrophy by siRNA transfection and Ang II induction. FTO knockdown reduced elevated hypertrophy indices induced by Ang II; this was also reflected by decreased mRNA expression levels of α-MHC/β-MHC/Serca-2a as shown by qPCR (Fig. 1B) and a reduction in the cell surface area of cardiomyocytes as shown by α-actinin staining (Fig. 1C). Ang II treatment increased mRNA (Fig. 1B) and protein (Fig. S3A) expression levels of FTO, which was suppressed by siFTO transfection. This finding indicated that FTO is essential in cardiomyocyte hypertrophy, and the inhibition of FTO expression would be beneficial for alleviating hypertrophy.

For further verification of the function of FTO in cardiac hypertrophy *in vivo*, AAV9 virus was injected through the tail vein to reduce FTO expression. As predicted, the protein expression of FTO was distinctly elevated in the myocardial tissues of the TAC + shNC group compared with that in the Sham + shNC group, while it was inhibited in the TAC + shFTO group (Fig. S3B). In comparison with that in the Sham + shFTO group, FTO expression in the TAC + shFTO group was slightly elevated; however, the difference was not significant, which might be because of a low expression of FTO in myocardial tissues due to shFTO treatment. In the TAC + shNC group, TAC surgery caused myocardial hypertrophy in mice, which was apparently reflected by an increase in heart size (including heart-to-body weight ratio), cell surface area, and fibrosis. However, FTO knockdown (TAC + shFTO group) prevented this change, wherein a decrease occurred in heart size, cell surface area, and collagen fiber in the TAC + shFTO group compared with those in the TAC + shNC group (Fig. 1D, E). In contrast, TAC + shNC mice exhibited marked cardiac dysfunction, which was reversed by FTO knockdown, as demonstrated by a decrease in left ventricular posterior wall thickness at end-diastole and at end-systole in the TAC + shFTO group (Fig. 1F). In addition, TAC surgery or shFTO virus injection did not make any differences on ejection fraction and fractional shortening (Fig. 1G), which indicated the presence of normal mouse cardiac function. These findings suggested that FTO knockdown relieved cardiac hypertrophy *in vivo.*

To clarify the molecular mechanism through which FTO regulates cardiac hypertrophy, we conducted MeRIP sequencing in cardiomyocytes. We assessed only two differentially expressed genes, namely *OBSCN* and *CHD7* (chromodomain helicase DNA-binding protein 7), in different groups of cells. Conditional screening with MeRIP sequencing and mRNA sequencing revealed a low level of m^6^A methylation and mRNA expression in the Ang II + siNC group, while an elevated level in the Ang II + siFTO group for both these genes (Fig. S3C).

Next, qPCR was conducted with RNA extracted from the myocardial tissues of TAC-treated mice to confirm the mRNA expression of OBSCN and CHD7. As shown in Figure 1H, the mRNA expression of OBSCN was increased in the Sham + shFTO group compared with that in the Sham + shNC group. Moreover, OBSCN mRNA expression was reduced in the TAC + shNC group but was elevated in the TAC + shFTO group; however, a similar result was not observed for CHD7. The mRNA expression of FTO was consistent with its protein expression in mice. We also conducted MeRIP-qPCR to measure the m^6^A abundance of OBSCN and CHD7 mRNAs in the myocardial tissues of TAC-treated mice. The results indicated that the m^6^A methylation level of *OBSCN* was decreased in the TAC + shNC group and increased in the Sham + shFTO group; MeRIP sequencing showed a similar result for *OBSCN*, but a different result for the m^6^A methylation level of *CHD7* (Fig. 1I). The *OBSCN* gene spans more than 150 kb in length; therefore, we performed transfection of cardiomyocytes with a siRNA for OBSCN. The knockdown of *OBSCN* promoted the mRNA expression of hypertrophy indices (α-MHC/β-MHC/Serca-2a) (Fig. 1J); this finding indicated that *OBSCN* could suppress cardiomyocyte hypertrophy. Overall, we considered that FTO down-regulation inhibited the mRNA expression of OBSCN by potentially influencing the m^6^A methylation sites to relieve hypertrophy (Fig. 1K).

In summary, our results suggest that FTO-mediated m^6^A demethylation plays a critical role in myocardial function and FTO knockdown in mice significantly improved myocardial hypertrophy. Further studies on the relationship between FTO and various cardiovascular diseases may provide a novel therapeutic approach to prevent and treat various cardiovascular diseases.

## Ethics declaration

All animal experiments were approved by the Animal Ethics Committee of Soochow University.

## Conflict of interests

The authors declare no conflict of interests.

## Funding

This work was financially supported by the National Natural Science Foundation of China (No. 82100251).
