# Supplementary material for: Effect of FTO on cardiac hypertrophy through the regulation of OBSCN expression
Source: Genes Dis. 2023 Nov 11;11(6):101165. doi: 10.1016/j.gendis.2023.101165 (PMC11327526; doi:10.1016/j.gendis.2023.101165)
Supplement: Multimedia component 1 [file mmc1.docx]

**Effect of FTO on cardiac hypertrophy through the regulation of *OBSCN* expression**

Lili Chen, Yiheng Zhao, Wenjing Wang, Shuchen Zhang, Xiang Zhou

**Supplementary Methods**

**Cell culture and transient transfection**

Isolation of mouse cardiomyocytes was performed on the myocardial tissues from 1- to 3-day-old newborn C57BL/6J mice. Briefly, the obtained cells were resuspended in 10% fetal bovine serum (FBS)-supplemented DMEM and pre-plated on a 6-cm dish for 1.5 h to remove fibroblasts. Nonadherent cardiomyocytes in DMEM adding 10% FBS and 1% 5-bromodeoxyuridine were plated and then treated with 1 μM Ang II for 24 h to generate the hypertrophic phenotype *in vitro*.

The transfection of cardiomyocytes was achieved by Lipofectamine™ 3000 reagent (Invitrogen, Carlsbad, CA, USA) with 50 nM siRNA for *FTO* or *OBSCN*. siRNA for *FTO* and *OBSCN* were synthesized by GenePharma (Shanghai, China). The used siRNA sequences were as below: *FTO* siRNA, 5′-AACAAAGGAGUGAGAUUCUTT-3′; *OBSCN* siRNA, 5′-GGUACGAGAGAAAGAGUCUTT-3′; NC siRNA, 5′-UUCUCCGAACGUGUCACGUTT-3′.

**Reverse transcription and qPCR analyses**

The extraction of total RNA from frozen cardiac tissues or cardiomyocytes was performed with TRIzol reagent (Invitrogen). The RNA extract (2000 ng) was then reverse transcribed to obtain first-strand cDNA by using HiScript^®^ III RT SuperMix for qPCR (+gDNA wiper) (Vazyme, Nanjing, China). qPCR was conducted on a CFX96 real-time PCR detection system (BioRad, CA, USA). The primer sequences for FTO, OBSCN, ANP, α-MHC, β-MHC, and Serca-2a were as below: FTO, forward: 5ʹ- GCAGCTGAAATACCCTAAACTG-3ʹ and reverse: 5ʹ- AGTCTGGTGTTCAAGTACTTGT-3ʹ; OBSCN, forward: 5ʹ-AGATGTTCTGTGCCGCCAAGTTC-3ʹ and reverse: 5ʹ-GTGTAGCCAAGATGTCTCGTTCCTG-3ʹ; CHD7, forward: 5ʹ-CGACAATGAAGACGAGAACCGAGAC-3ʹ and reverse: 5ʹ-GCAGTAGCAGCACCAACAGATCC-3ʹ; ANP, forward: 5ʹ-CCAGCATGGGCTCCTTCTCCA-3ʹ and reverse: 5ʹ-CCGGAAGCTGTTGCAGCCTAGT-3ʹ; α-MHC, forward: 5ʹ-CTACCAGACAGAGGAAGACAAG-3ʹ and reverse: 5ʹ-TAGGCCTTCACCTTCAACTGTA-3ʹ; β-MHC, forward: 5ʹ-CCGAGTCCCAGGTCAACAA-3ʹ and reverse: 5ʹ-CTTCACGGGCACCCTTGGA-3ʹ; Serca-2a, forward: 5ʹ-TTCTGCTTATCTTGGTAGCCAA-3ʹ and reverse: 5ʹ-CTTTCTGTCCTGTCGATACACT-3ʹ; GAPDH, forward: 5ʹ-AAGGTCATCCCAGAGCTGAA-3ʹ and reverse: 5ʹ-CTGCTTCACCACCTTCTTGA-3ʹ.

**MeRIP-qPCR**

For the quantification of *OBSCN* m^6^A RNA methylation level, total RNA was extracted from mouse myocardial tissues. MeRIP was executed with the Magna MeRIP m^6^A kit following the instructions of the manufacturer (#17-10499, Millipore, MA, USA). Briefly, a small fraction of the RNA sample was separated as the input after fragmenting the RNA. The remaining RNA was then immunoprecipitated by incubating with m^6^A antibody-conjugated beads at 4 °C overnight to obtain RNA samples with m^6^A, which were rinsed from the beads and further purified. For input samples and m^6^A-immunoprecipitated RNA samples obtained as mentioned above, qPCR was conducted for measuring the mRNA expression levels of genes.

**m^6^A** **RNA sequencing**

Cardiomyocytes were collected and lysed in TRIzol reagent (Invitrogen). The quantification of total RNA was performed using NanoDrop ND-1000 (Thermo Fisher Scientific, Waltham, MA, USA), and 100-nucleotide-long fragments were obtained by chemically fragmenting. MeRIP was conducted as mentioned above. First, a HiSeq sequencer (Illumina) was used to sequence the samples for 150 cycles after denaturation into single-stranded RNA molecules, capture on Illumina flow cells, and amplification *in situ* as clusters in accordance with the manufacturer’s instructions. Next, image analysis and base calling were performed by using Solexa pipeline (Off-Line Base Caller software, version 1.8). FastQC software was used to check sequencing quality, while HISAT2 software (version 2.1.0) was used to trim the sequences (illumina quality filter and cutadapt pruning adapter base) to compare the sequencing results with the genome sequence in the Ensembl database. We then identified the MeRIP-enriched regions (peaks) of each transcript which were annotated using overlapping genes from the latest version of the Ensembl database, and used exon peaks for comparison. The statistical analysis for m^6^A peaks of each transcribed region was then proceeded. For RNA sequencing, mRNA expression levels of gene were measured with the m^6^A-seq input data. The mouse genome database (GRCm38) was used to map the trimmed reads to the corresponding reference genome with HISAT2 software. The reconstruction of the transcriptome was accomplished using StringTie. Ballgown software was used for the calculation of the differential expression levels.

**Western blotting assay**

Cardiomyocytes or myocardial tissues were lysed in RIPA lysis buffer (Beyotime, Shanghai, China) to extract total proteins. Immunoblot assays were performed following the standard protocols. Polyvinylidene difluoride membranes were used to transfer proteins separated by SDS-PAGE. Quantitative analysis was accomplished by using ImageJ software. Primary antibodies of anti-FTO (ab126605, Abcam Cambridge, MA, USA), anti-GAPDH (60004-1, Proteintech, Hubei, China), and anti-mice or anti-rabbit secondary antibodies (Multi Science, Hangzhou, China) were commercially purchased.

**Measurement of cell surface area**

The cell surface area of cardiomyocytes was measured by immunofluorescence following staining with actinin. Cardiomyocytes were immersed in 4% formaldehyde to immobilize cells for 30 min, treated with 0.1% Triton X-100 at 4 °C for 10 min, and then incubated with 5% bovine serum albumin in PBS for 1 h. Next, the anti-α-actinin antibodies (5 μg/mL, Abcam) in PBS was incubated with cells at 4 °C overnight and immediately incubated with Alexa 594-conjugated secondary antibodies at room temperature for 1 h. The images acquisition was conducted on laser confocal microscopy (LSM800, Zeiss, Oberkochen, Germany).

**Mouse model of cardiac hypertrophy**

A mouse model of myocardial hypertrophy was constructed by transverse aortic constriction (TAC) surgery. Eight- to 10-week-old male C57BL/6J mice were used for model construction and were anesthetized by administering a mixed anesthetic agent containing ketamine and xylazine (100 mg/kg and 2.5 mg/kg, respectively). Adeno-associated virus 9 (AAV9) was injected through the mouse tail vein to reduce FTO expression at the 3rd days after TAC. Briefly, AAV9-shFTO (1 × 10^11^) and AAV9-shNC (1 × 10^11^) were injected into TAC-treated mice (TAC group) and control mice (Sham group), respectively; the groups were designated as the TAC+shFTO group and the Sham+shFTO group, respectively. Measurements of echocardiography were conducted on all mice in a blinded manner. The following analyses were conducted at 4 weeks after TAC or Sham operation: left ventricular posterior wall thickness at end-diastole (LVPWd), LV posterior wall thickness at end-systole (LVPWs), ejection fraction (EF%) and fractional shortening (FS%).

**Histological analysis**

Heart tissue samples were immersed in 4% formaldehyde for immobilization, then embedded in paraffin or an OCT compound for sectioning. The measurement of cell surface areas was performed by staining with fluorescein isothiocyanate-conjugated wheat germ agglutinin (WGA, Sigma Chemical, St. Louis, MO, USA) on frozen sections. For histopathological analysis, paraffin-embedded sections were stained with hematoxylin and eosin (H&E) and Masson’s trichrome stain (Solarbio, Beijing, China) following the instructions of the manufacturer and viewed under a microscope.

For m^6^A staining, cardiomyocytes were fixed with 4% formaldehyde in PBS, incubated with anti-m^6^A antibodies (2 μg/mL, Abcam) at 4 °C overnight, along with the incubation with Alexa 594-conjugated secondary antibodies at room temperature for 1 h. The images acquisition was obtained by using laser confocal microscopy.

**Quantification of m^6^A RNA methylation**

RNA was extracted from cardiomyocytes. The extracted RNA samples were proceeded to m^6^A quantification by using the EpiQuik m^6^A RNA methylation quantification kit (P-9005, EpiGentek, Farmingdale, NY, USA) in accordance with the manufacturer’s protocol.

**Statistical analysis**

All results are expressed as mean ± SEM. Differences among groups were evaluated using Student’s t-test (for two groups) or one-way analysis of variance (ANOVA) (for more than two groups). Statistical analysis was performed using GraphPad Prism 6.0. *P*-values <0.05 were regarded significant.

**Supplementary Figures**


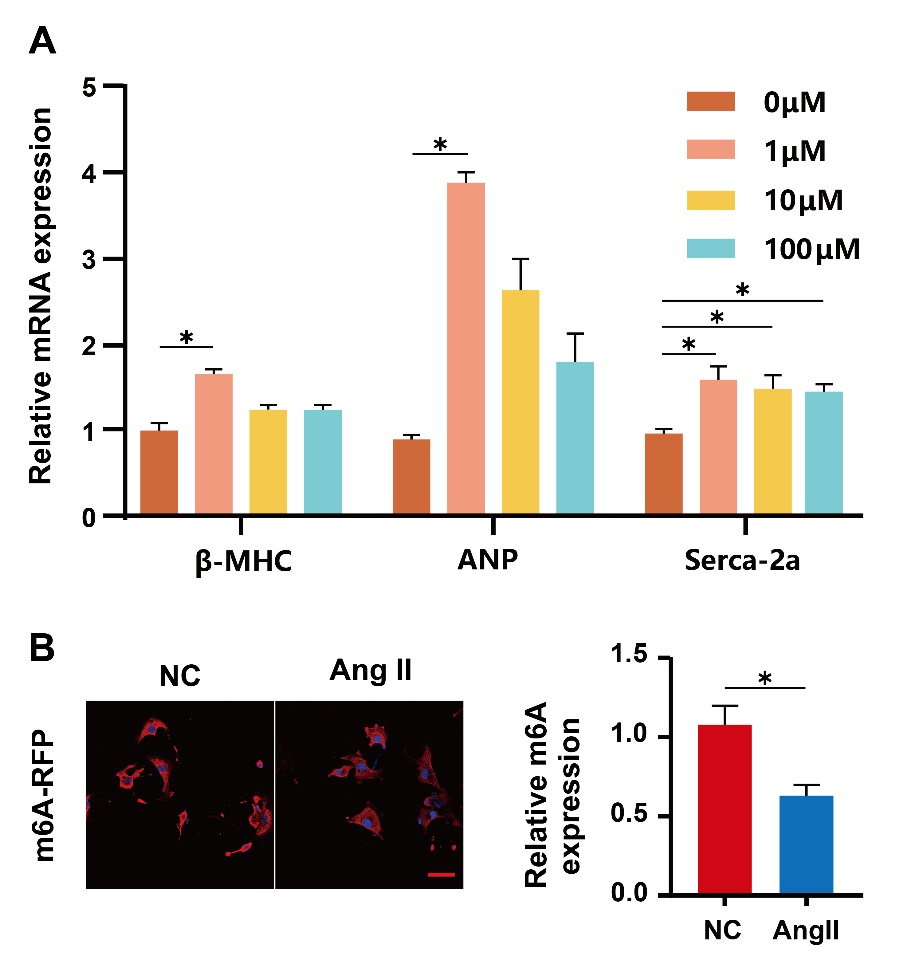


**Figure S1. m6A methylation levels were decreased** **in hypertrophic cardiomyocytes induced by AngII. (A)** Relative mRNA levels of β-MHC, ANP and Serca-2a in cardiomyocytes with different concentrations Ang II (0 μM, 1 μM,10 μM,100 μM) treatment. **(B)** Immunofluorescence detection of m^6^A in control cardiomyocytes and Ang II-induced (1 μM) cardiomyocytes. Scale bar, 20 μm. **P* < 0.05.


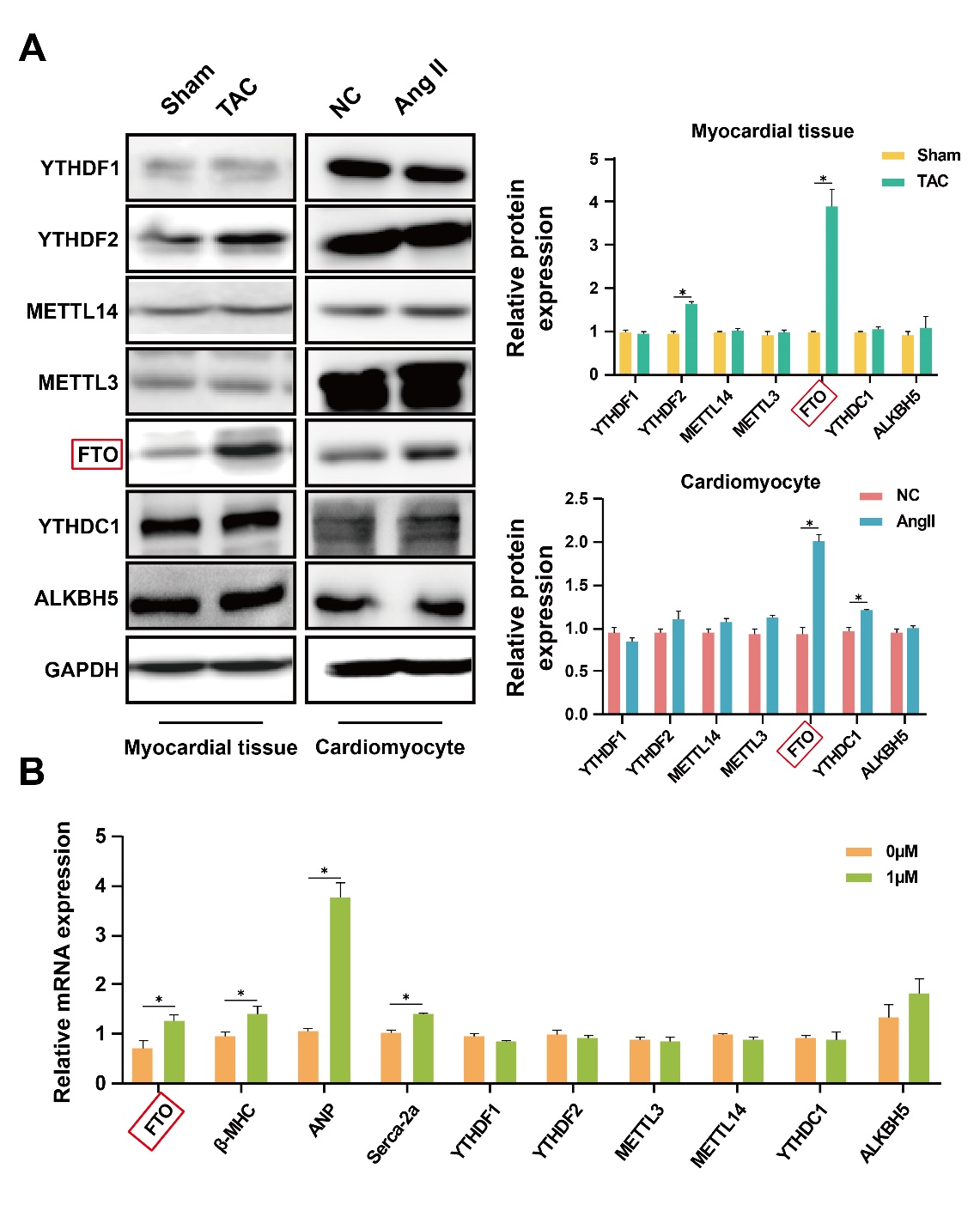


**Figure S2.** **FTO was overexpressed in hypertrophic cardiomyocytes and myocardial tissues of TAC-treated mice. (A)** Left: Western blotting assay of YTHDF1, YTHDF2, METTL14, METTL3, FTO, YTHDC1, and ALKBH5 in cardiomyocytes and myocardial tissues. Right: Relative protein levels of YTHDF1, YTHDF2, METTL14, METTL3, FTO, YTHDC1, and ALKBH5 in cardiomyocytes and myocardial tissues. **(B)** Relative mRNA levels of FTO, β-MHC, ANP, Serca-2a, YTHDF1, YTHDF2, METTL3, METTL14, YTHDC1, and ALKBH5 in cardiomyocytes. FTO: Fat and obesity-associated protein; YTHDF1:  YTH N6-methyladenosine RNA binding protein F1; YTHDF2: YTH N6-methyladenosine RNA binding protein F2; METTL14: Methyltransferase 14; METTL3: Methyltransferase 3; YTHDC1:  YTH N6-methyladenosine RNA binding protein C1; ALKBH5: AlkB homolog 5; TAC: Transverse aortic constriction; m^6^A: *N*^6^-methyladenosine. **P* < 0.05.


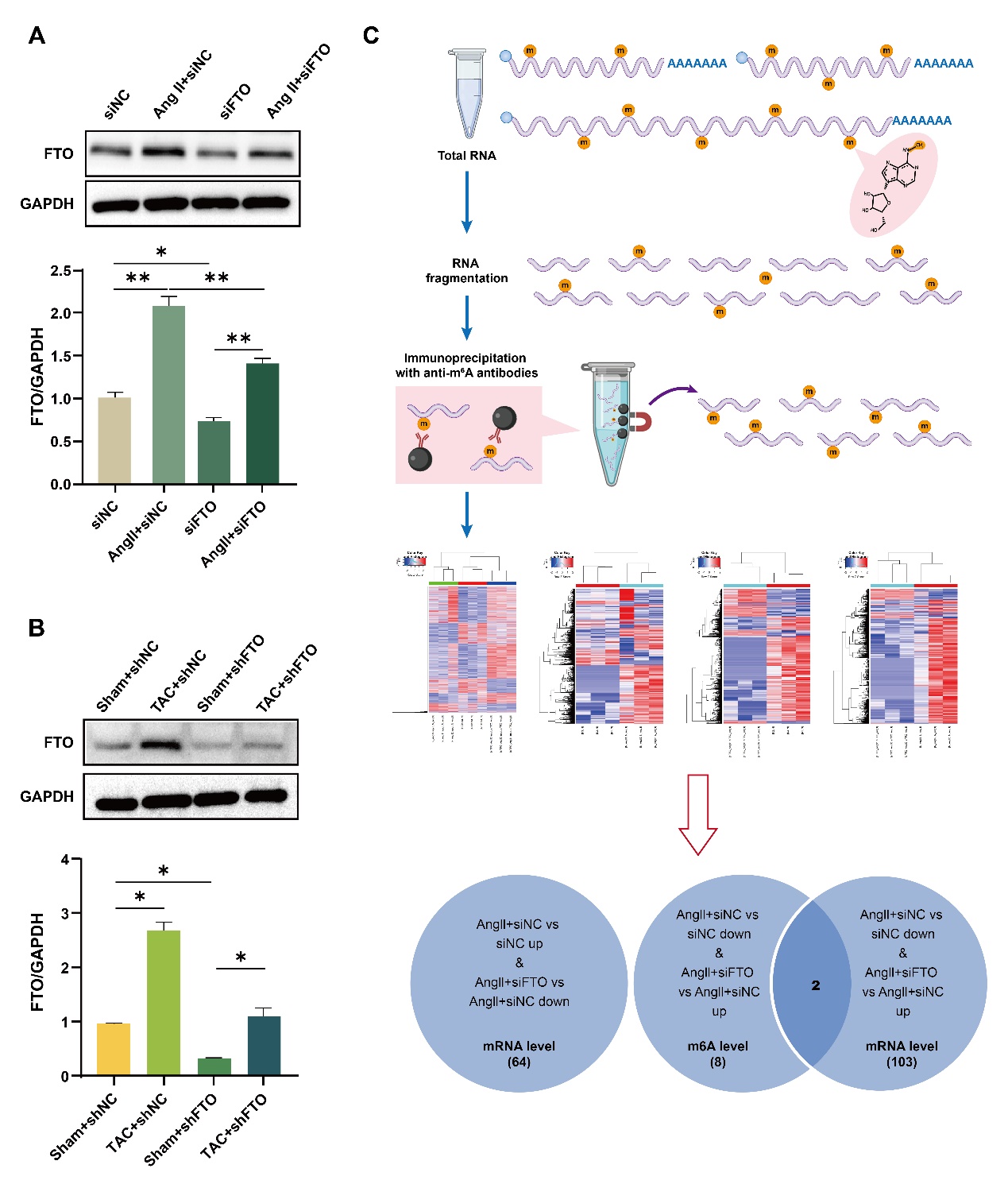


**Figure S3. Expression of FTO was detected in** **cardiomyocytes and myocardial tissues. (A)** Western blotting assay of FTO and relative protein expression of FTO in cardiomyocytes induced by 1 μM Ang II and/or transfected with siFTO. **(B)** Western blotting assay of FTO and relative protein expression of FTO in myocardial tissues of mice. **(C)** MeRIP-seq was conducted in cardiomyocytes treated with 1 μM Ang II and/or siFTO transfection. **P* < 0.05; ***P* < 0.01.
